# Association between Proton Pump Inhibitor Use and CNS Infection Risk: A Retrospective Cohort Study

**DOI:** 10.3390/jcm7090252

**Published:** 2018-08-31

**Authors:** Wei-Te Hung, Ying-Hock Teng, Shun-Fa Yang, Han-Wei Yeh, Ying-Tung Yeh, Yu-Hsun Wang, Ming-Yung Chou, Ming-Chih Chou, Chi-Ho Chan, Chao-Bin Yeh

**Affiliations:** 1 Institute of Medicine, Chung Shan Medical University, Taichung 40201, Taiwan; cshy029@csh.org.tw (W.-T.H.); ysf@csmu.edu.tw (S.-F.Y.); mcchou@gmail.com (M.-C.C.); 2 School of Medicine, Chung Shan Medical University, Taichung 40201, Taiwan; 3 Department of Anesthesiology, Chung Shan Medical University Hospital, Taichung 40201, Taiwan; 4 Department of Emergency Medicine, School of Medicine, Chung Shan Medical University, Taichung 40201, Taiwan; cshy392@csh.org.tw; 5 Department of Emergency Medicine, Chung Shan Medical University Hospital, Taichung 40201, Taiwan; 6 Department of Medical Research, Chung Shan Medical University Hospital, Taichung 40201, Taiwan; cshe731@csh.org.tw; 7 School of Medicine, Chang Gung University, Taoyuan City 33302, Taiwan; george66889@gmail.com; 8 Graduate School of Dentistry, Chung Shan Medical University, Taichung 40201, Taiwan; yehtungtung@hotmail.com; 9 School of Dentistry, Chung Shan Medical University, Taichung 40201, Taiwan; myc@csmu.edu.tw; 10 Department of Dentistry, Chung Shan Medical University Hospital, Taichung 40201, Taiwan; 11 Department of Microbiology and Immunology, Chung Shan Medical University, Taichung 40201, Taiwan

**Keywords:** proton pump inhibitors, CNS infection, retrospective cohort study

## Abstract

This study investigated the incidence of central nervous system (CNS) infection following the use of proton pump inhibitors (PPIs). A retrospective cohort study was conducted in Taiwan by using data from the National Health Insurance Research Database. We identified and enrolled 16,241 patients with CNS infection who used PPIs (PPI users). The patients were individually propensity score matched (1:1) according to age, sex, hypertension, hyperlipidemia, Charlson comorbidity index (CCI), H2 blocker, non-steroidal anti-inflammatory drugs (NSAIDs), corticosteroid, and immunosuppressant use with 16,241 controls (PPI nonusers). A Cox proportional hazards model was used to estimate adjusted hazard ratio (aHR) for CNS infection in the PPI users and nonusers. After adjustment for other confounding factors, the incidence of CNS infection in the PPI users was 2.23-fold higher than that in the PPI nonusers (95% CI = 1.27–3.94). In addition, the PPI users exhibited a higher risk of CNS infection than the nonusers in the hypertension and CCI = 1 groups (aHR = 3.80, 95% CI = 1.40–10.32; aHR = 2.47, 95% CI = 1.07–5.70 in the PPI users and nonusers, respectively). In conclusions, according to these results, we concluded that the incidence of CNS infection was higher in the PPI users than in the nonusers.

## 1. Introduction

Proton pump inhibitors (PPIs) have been clinically used for nearly three decades for treating patients of all ages [1,2]. To date, PPIs have been used for treating peptic ulcer disease and acid-secretion-related diseases including esophagitis and nonerosive reflux disease; for preventing ulcers associated with nonsteroidal anti-inflammatory drugs [3]; and for treating Zollinger-Ellison syndrome [4] and functional dyspepsia [2]. PPIs are also employed in combination therapy for treating *Helicobacter pylori* infections. PPIs are a family of structurally related prodrugs that inhibit the H^+^/K^+^-ATPase enzyme in parietal cells by covalently binding to the cysteine residue of the enzyme [3,5]. In addition to their action in the stomach, PPIs were recently reported to inhibit proton pumps for maintaining the pH level in the pancreas [6].

Observational studies have indicated that the widespread use of PPIs increases the risk of several systemic diseases. For example, patients using PPIs exhibited significantly higher rates of cardiovascular morbidity, all-cause mortality [7], and chronic kidney disease [8] than did PPI nonusers. In addition, PPIs increased the risks of *Clostridium difficile* infections [9], community-acquired enteric infections [10], and community-acquired pneumonia [11,12]. Moreover, PPI use has been proposed to increase the risk of central nervous system (CNS) diseases. For example, PPIs block the V-ATPase enzyme and modify the pH level in microglial lysosomes; consequently the long-term consumption of PPIs might be a risk factor for Alzheimer disease (AD) [13]. The accumulation of amyloid-species, a key feature of AD, was observed after the administration of lansoprazole in both cell cultures and animal models [14]. The administration of PPIs also increased risk of CNS diseases such as dementia [15] and hepatic encephalopathy [16]. Many pathogens, including a wide spectrum of bacteria, viruses, fungi, and parasites, can cause CNS infection [17]. In addition to pathogens, noninfectious factors such as age, occupation, immune status, sexual behavior, and drug abuse are key factors that increase the risk of CNS infection [18]. While PPIs were proved to cross the blood-brain barrier, many studies reported PPIs might suppress innate immunity [14,19,20,21,22]. Taken together, it is rationale to speculate that PPIs users may have higher risk to acquire CNS infection. However, the association between PPI usage and CNS infection remains unclear. This study investigates the risk of CNS infection following the use of PPIs. We hypothesized that PPI users are at a higher risk of CNS infection than are PPI nonusers.

## 2. Experimental Section

### 2.1. Study Population

The National Health Insurance (NHI) Research Database (NHIRD) was obtained from the Taiwan National Health Research Institutes. More than 99% of the entire population of Taiwan (23 million) is covered by the NHI program. One million beneficiaries were randomly sampled from the database. The distribution of age and sex in the sample did not differ from that of the entire population. The database contains information regarding all outpatient and inpatient medical claims, including medications, medical operations, procedures, and fees. The study was approved by the ethical review board of Chung Shan Medical University Hospital (CSMU No.: 18096).

### 2.2. Study Design

This study had a retrospective cohort design. The PPIs considered in this study were omeprazole, pantoprazole, lansoprazole, esomeprazole, and rabeprazole, which are available in Taiwan. New PPI users were recruited between 1 January 2010, and 31 December 2012. The first date of PPI use was considered the index date. To reduce immortal time bias, we defined PPI users as patients who used PPIs for at least 90 days within 1 year. Furthermore, we selected patients who were 20 years or older. Patients with a diagnosis of CNS infection before the index date were excluded. The PPI nonuser group comprised patients who did not use PPIs from 2009 to 2013. For both groups to have the same starting date, initially, a 1:10 ratio was used for age and sex matching while establishing an index date for the PPI nonuser group that corresponded to the index date of the PPI user group. The PPI nonuser group comprised patients who did not use PPIs from 2009 to 2013. Initially, a 1:10 ratio was used for age and sex matching while establishing an index date for the PPI nonuser group that corresponded to the index date of the PPI user group. During matching, we again excluded patients with diagnoses of CNS infection before the index date. Propensity score matching (1:1) was then performed for the PPI user and nonuser groups according to age, sex, hypertension (International Classification of Diseases, Ninth Revision, Clinical Modification [ICD-9-CM] codes 401-405), hyperlipidemia (ICD-9-CM codes 272.0-272.4), Charlson comorbidity index (CCI), and H2 blocker, non-steroidal anti-inflammatory drugs (NSAIDs), corticosteroid and immunosuppressant use. The propensity score was estimated by the logistic regression. The PPI user and the PPI nonuser were the binary outcome. By matching the propensity score, we could determine similarities among both groups. All comorbidities were defined if diagnoses had been made at least 1 year before the index date in at least two outpatient visits or one admission. The use of H2 blocker, NSAIDs, corticosteroid, and immunosuppressant use were defined as use for 90 days or more during the study period. To test the goodness-of-fit, the c-statistic was 0.50. It represents consistency between the two groups. The propensity score distribution was showed in Supplementary Figure S1. To validate the consistency of the results, we also performed an analysis of before propensity score matching (PSM) matched (Supplementary Table S1).

### 2.3. Outcome Measurement

The outcome was defined as a newly diagnosed CNS infection (ICD-9-CM codes 320-324) after the index date. Both the (PPI user and nonuser) groups were followed up until the first date of CNS infection, withdrawal from the NHI program, or the end of 2013, whichever came first.

### 2.4. Statistical Analysis

To compare the characteristics of the PPI user and nonuser groups, the Student t test or Chi-squared test were used as appropriate. Continuous variables are presented using means and standard deviations. Categorical variables are presented using numbers and percentages. Kaplan-Meier analysis was used to compare the cumulative incidence of CNS infection between the PPI user and nonuser groups. Statistical significance was determined using the log-rank test. The independent risk of the PPI group was determined and hazard ratios were estimated using the multivariate Cox proportional hazards model. SPSS Version 18.0 (SPSS, Chicago, IL, USA) was used for statistical analysis. Values of *p* < 0.05 were considered statistically significant.

## 3. Results

### 3.1. Characteristics of the Study Cohort

**Figure 1:**
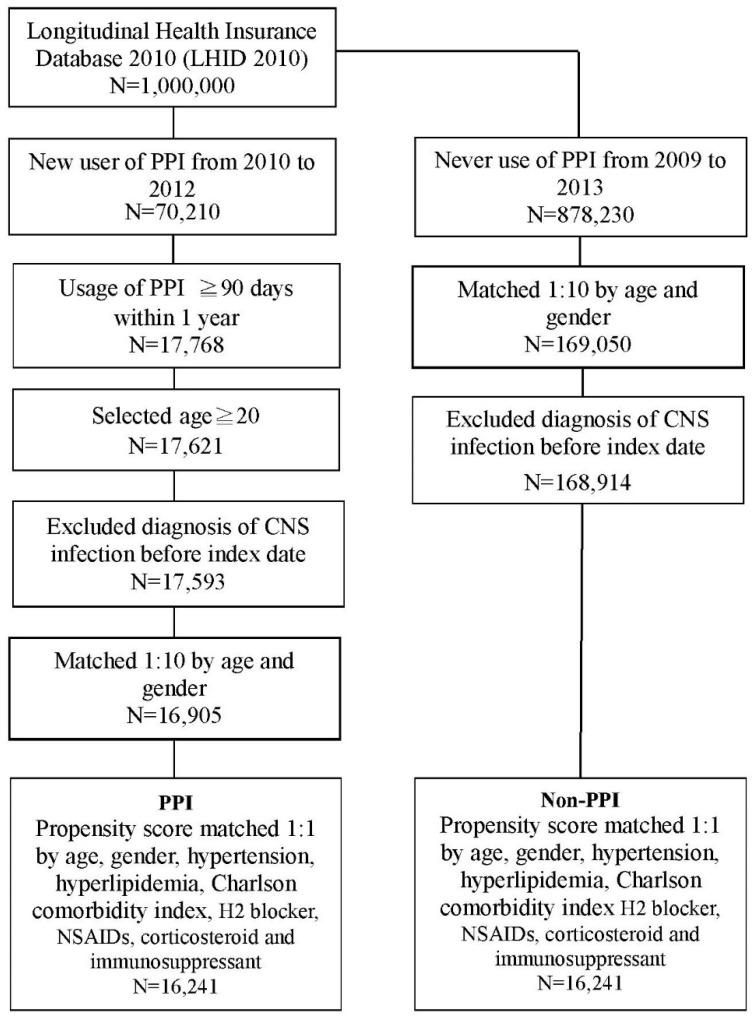
Flow chart for PPI patient’s selection. PPI, proton pump inhibitors; CNS, central nervous system; NSAIDs, non-steroidal anti-inflammatory drugs.

In total, 70,210 new PPI users between 2010 and 2012 were included in this study. Patients with diagnosis of CNS infection before the index date were excluded (*n* = 17,593). Next, we performed (1:10) age and sex matching to determine an index date for the PPI nonusers corresponding to that of the PPI user group. Finally, 16,241 PPI users and an equal number of PPI nonusers were further screened through propensity score matching for age, sex, comorbidities, CCI, and H2 blocker, NSAIDs, corticosteroid, and immunosuppressant use (Figure 1). Both groups (PPI user and nonusers) exhibited similar numbers and percentages in terms of age, sex, comorbidities, severity of comorbidities, and drugs use (Table 1).

### 3.2. Risk of CNS Infection in PPI Users

**Figure 2:**
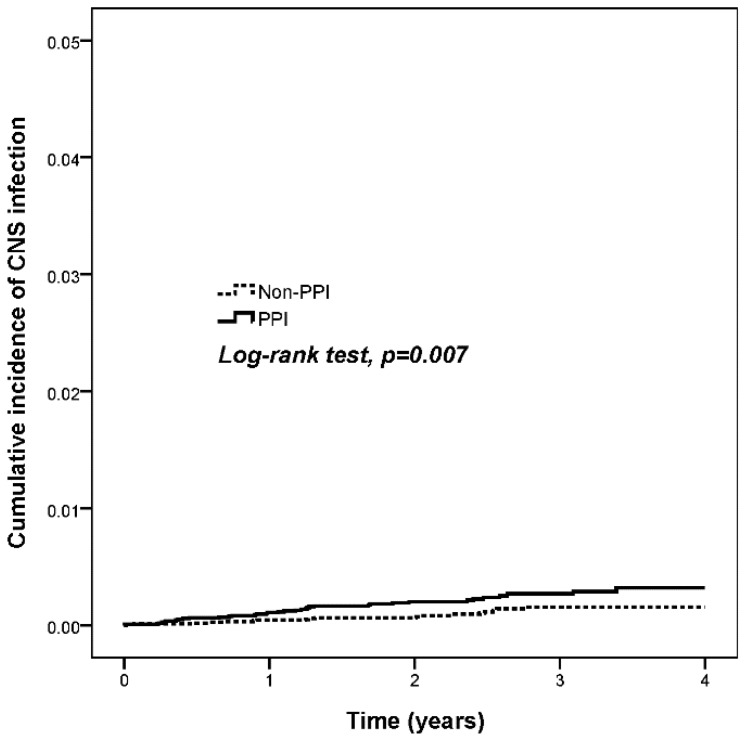
Kaplan-Meier curves of the occurrence of CNS infections in PPI and non-PPI usage patients. PPI, proton pump inhibitors; CNS, central nervous system.

We next estimated the cumulative incidence of CNS infection in the PPI users and nonusers. The Kaplan-Meier curve representing the PPI users depicts a visibly higher incidence of CNS infection in the PPI users than in the nonusers for 4 years (log-rank test, *p* = 0.007) (Figure 2). According to the Cox proportional hazards model, the incidence of CNS infection in the PPI users was 2.23-fold higher than that in the PPI nonusers (Table 2). Among the patients with CCI score ≥2, the adjusted hazard ratios (HRs) revealed that the PPI users exhibited a 3.07-fold higher severity of CNS infection than the nonusers did. By contrast, no correlation was observed between H2 blocker use and CNS infection (Table 2).

### 3.3. Risk of CNS Infection in PPI Users and Nonusers and Subgroup Specific Characteristics

We conducted subgroup analysis between the PPI users and nonusers (Table 3). The risk of CNS infection was notably higher in the PPI-using patients aged 40 to 65 years (2.23-fold) and ≥65 years (3.23-fold) than in their non-PPI-using counterparts. The risk of CNS infection was also significantly higher in the male PPI user group than in the male nonuser group (HR = 2.39). Similarly, the PPI users exhibited a higher risk of CNS infection than the nonusers in the hypertension and CCI = 1 groups. No correlation between PPI use and CNS infection was observed in the H2 blocker group.

## 4. Discussion

PPIs have been commonly used for treating gastric and related diseases for three decades. The widespread use of PPIs has been found to be a risk factor for several infectious and systemic diseases. An updated literature review indicated that PPI therapy was strongly associated with the development of community-acquired enteric infections, particularly those caused by *Salmonella* spps. and *Campylobacter* spps. [10]. Other enteric diseases, including *Clostridium difficile* infections, were also reported to be associated with the use of PPIs [9]. In fact, a study conducted in the past decade indicated that patients who received omeprazole for a short period exhibited overgrowth of some bacteria in the duodenum [23]. The most rational pathogenesis was that PPIs modified the pH level in the digestive system, which might have facilitated the growth of several enterobacteria [24]. Apart from research associating infections of the digestive system with PPI use, a meta-analysis of studies on PPIs was conducted and revealed that the overuse of PPIs might increase the risk of chronic kidney disease [8] and cardiovascular events, which result in higher likelihood of mortality [7]. Our results, which showed that the PPI users had a 2.23-fold higher risk of CNS infection than the PPI nonusers (95% CI = 1.27–3.94), are similar to those of the meta-analysis. Despite the neurotropic pathogenicity of pathogens, several risk factors have been identified to increase the susceptibility of the pathogens to infect CNS, e.g., community-acquired bacterial meningitis [25]. These factors consist of medical conditions resulting in immunodeficiency (i.e., splenectomy), host genetic factors (i.e., polymorphism of genes involving innate immunity) or anatomical defects of the natural barriers of the CNS. Other risk factors including socioeconomic factors, age, genetic variation of the host, and underlying medical conditions and diseases associated with increased susceptibility to invasive bacterial infections in both children and adults [26,27]. In our study, PPIs usage is another risk factor for developing CNS infection. Hence, our study demonstrated that PPI users have higher risk of CNS infection than do PPI nonusers.

PPIs can affect the CNS and increase the risk of CNS-related diseases. PPI use was proposed to be a predisposing factor for AD. Animal experiments demonstrated the ability of PPIs to cross the blood–brain barrier [13]. PPIs bind to the V-ATPase proton pump on the surface of microglial cells, basify the lysosomes of microglial cells, and alter the phagocytic ability of these cells; thus, PPIs adversely affect the clearance of amyloid-β species by microglial cells. The long-term use of PPIs was proposed to be a predisposing factor for AD [13]. Furthermore, one PPI, lansoprazole, was shown to enhance the production of amyloid-β species in both in vitro and in vivo models [14]. Thus, there were several studies related to PPIs and dementia in the following years. Haenisch & colleagues examined the effect of PPIs on the risk of dementia in elderly patients (age ≥ 75) [28]. Patients receiving PPI medication had a significantly increased risk of any dementia. Unfortunately, it is difficult to know whether different types of PPIs had a different level of risk of dementia on elderly patients. Meanwhile, an extensive reviews of 1055 relevant articles were analyzed and was concluded that there was an increased risk of dementia among PPIs users [29]. An increased risk for dementia was also identified among the Taiwanese PPI users. The risk was increasing among different age groups from 28–48, 49–83, and age ≥83 [30]. By contrast, observational studies revealed that the use of PPIs might reduce the risk of mild cognitive impairment and dementia [15,31]. A most up to date study indicated that exposure to H2RAs, but not PPIs, was associated with increased dementia risk in Korean population [32]. In fact, many reported association of dementia and PPIs were still limited by their different methodology and conflicting results [33]. A meta-analysis investigated whether PPIs increased the risk of other CNS diseases such as hepatic encephalopathy; however, the results were controversial [16]. Additional studies might be required to verify the effect of PPI use on dementia. In the present study, the risk of CNS infection was relatively high in male patients and those aged ≥65 years (3.12-fold, 95% CI = 1.12–8.67). Therefore, old age and male sex are critical risk factors for CNS infection.

PPIs might suppress innate immune responses. Documented evidence shows that PPIs interfered with the functionality of neutrophils. For example, acid-activated omeprazole inhibited phagocytosis and acidified the phagolysosomes of human neutrophils in vitro; thus, omeprazole impaired neutrophil function [19]. The formation of PPI-peptide adducts might inhibit the activity of lysosomal enzymes and alter the enzyme functions [34]. Furthermore, rabeprazole, but not omeprazole or lansoprazole, was demonstrated to reduce the chemotaxis of human neutrophils [20]. An experiment with human volunteers showed that healthy individuals who received only a single dose of omeprazole exhibited diminished production of intracellular and extracellular reactive oxygen species by neutrophils; consequently, the bactericidal activity of the neutrophils was diminished [21]. A recent study demonstrated that the use of PPIs reduced the cellular oxidative burst in granulocytes and monocytes, particularly in patients with cirrhosis [22]. Because neutrophils play a crucial role in host defense against infection, PPI treatment increases susceptibility to bacterial infection. Thus, patients receiving PPI medication have a high risk of CNS infection.

PPIs might also interfere with the functions of monocytes and their differentiated forms, such as macrophages, osteoclasts, and microglial cells, in different organs. In the past two decades, both omeprazole and lansoprazole have been shown to demonstrate inhibitory effects on DNA synthesis in peripheral mononuclear cells in in vitro experiments [35]. Short-term administration of lansoprazole in patients with gastric ulcers reduced the expression of intercellular adhesion molecule-1 on peripheral blood monocytes, thus reducing the chemotactic ability of the monocytes [36]. Recently, many studies have suggested that PPIs increased the risk of osteoporosis and bone fracture, particularly in elderly patients [37,38,39]. PPIs are believed to impair the function of osteoclasts, a subpopulation of macrophages in the bone. Because the function of osteoclasts is critical in the maintenance, repair, and remodeling of bones, an interruption to osteoclast function might increase the risk of osteoporosis. Cell culture experiments demonstrated that omeprazole, esomeprazole, and lansoprazole induced apoptosis of human osteoclasts [40]. These findings were also supported by the results from another study, which showed that pantoprazole reduced cell viability and tartrate-resistant acid phosphatase activity in human osteoclasts in vitro [41]. Despite description of the inhibitory effects of PPIs on osteoclasts, few reports about the effects of PPIs on the antimicrobial activity of macrophages have been published. Only one report demonstrated that lansoprazole suppressed murine macrophage activity against *Candida* spp. [42]. Because microglial cells are the resident macrophages of the CNS, we can hypothesize that the antimicrobial activity of microglial cells is also diminished by PPI use; therefore, PPI use increases the risk of CNS infection.

Our study had some limitations. First, the database used did not contain information regarding clinical parameters, such as Glasgow Coma Scale scores and severity of CNS infection. Second, laboratory data and microbiological culture data that might have affected CNS infection occurrence were not included in this database. Third, we did not have access to potentially relevant personal behavioral information, such as smoking habit, alcohol consumption, and body mass index. These confounding factors might have affected the results. Fourth, Taiwan’s NHI system involves the Taiwanese population, and our data accurately reflect the situation in Taiwan. However, our results may not be applicable to Western populations.

## 5. Conclusions

In conclusion, PPI use is strongly associated with CNS infection. Moreover, the PPI users in the age groups 40–65, ≥65 and the male PPI users exhibited a relatively high risk of CNS infection. Physicians should exercise caution when prescribing PPIs to patients.

## Figures and Tables

**Table 1:** Demographic characteristics of PPI and Non-PPI.

	Unmatched					Matched				
	PPI (*N* = 16,905)		Non-PPI (*N* = 168,914)			PPI (*N* = 16,241)		Non-PPI (*N* = 16,241)		
	*n*	%	*n*	%	*p*-Value	*n*	%	*n*	%	*p*-Value
Age					1					0.144
20–40	2301	13.6	22,999	13.6		2123	13.1	2005	12.3	
40–65	9029	53.4	90,223	53.4		8557	52.7	8637	53.2	
≥65	5575	33.0	55,692	33.0		5561	34.2	5599	34.5	
Mean ± SD	57.8 ± 15.3		57.8 ± 15.3		0.977	58.2 ± 15.3		59.6 ± 14.9		<0.001 **
Gender					0.997					0.991
Female	8242	48.8	82,356	48.8		7911	48.7	7912	48.7	
Male	8663	51.2	86,558	51.2		8330	51.3	8329	51.3	
Hypertension	6579	38.9	45726	27.1	<0.001 **	6399	39.4	6393	39.4	0.946
Hyperlipidemia	3283	19.4	21,316	12.6	<0.001 **	3202	19.7	3165	19.5	0.605
CCI ^†^					<0.001 **					0.327
0	4618	27.3	122,220	72.4		4618	28.4	4592	28.3	
1	9146	54.1	35,275	20.9		8664	53.3	8780	54.1	
≥2	3141	18.6	11,419	6.8		2959	18.2	2869	17.7	
H2 Blocker	4354	25.8	6190	3.7	<0.001 **	3690	22.7	3683	22.7	0.926
NSAIDs	2309	13.7	11,587	6.9	<0.001 **	2165	13.3	2283	14.1	0.057
Corticosteroid	2576	15.2	7651	4.5	<0.001 **	2368	14.6	2457	15.1	0.165
Immunosuppressant	99	0.6	193	0.1	<0.001 **	77	0.5	74	0.5	0.807
Propensity score										
Mean ± SD	0.22 ± 0.2		0.08 ± 0.09			0.50 ± 0.01		0.50 ± 0.01		
Min, Max	0.02, 0.93		0.02, 0.91			0.47, 0.54		0.47, 0.53		

PPI, proton pump inhibitors; NSAIDs, non-steroidal anti-inflammatory drugs; ^†^ Charlson comorbidity index; ** *p* < 0.01.

**Table 2:** Cox proportional hazard model of CNS infection events.

	No. of CNS Infection Event	Observed Person-Years	Incidence Density (Per 1000 Person-Years)	Crude HR	95% CI	Adjusted HR ^‡^	95% CI
PPI							
No	18	41,444	0.4	1		1	
Yes	36	38,454	0.9	2.14 **	1.22–3.78	2.23 **	1.27–3.94
Age							
20–40	5	10,235	0.5	1		1	
40–65	30	42,944	0.7	1.43	0.56–3.69	1.25	0.47–3.32
≥65	19	26,718	0.7	1.46	0.54–3.90	0.95	0.32–2.83
Gender							
Female	25	39,057	0.6	1		1	
Male	29	40,841	0.7	1.11	0.65–1.89	1.08	0.63–1.84
Hypertension	22	31,116	0.7	1.08	0.63–1.85	0.91	0.50–1.66
Hyperlipidemia	8	15,831	0.5	0.70	0.33–1.49	0.63	0.29–1.35
CCI ^†^							
0	10	23,373	0.4	1		1	
1	26	43,469	0.6	1.40	0.67–2.90	1.36	0.64–2.89
≥2	18	13,056	1.4	3.21 **	1.48–6.95	3.07 **	1.33–7.08
H2 Blocker	13	19,341	0.7	1.00	0.54–1.87	0.82	0.42–1.57
NSAIDs	14	13,025	1.1	1.85 *	1.00–3.41	1.57	0.81–3.04
Corticosteroid	18	13,045	1.4	2.62 **	1.48–4.61	2.17 *	1.17–4.03

CNS, central nervous system; PPI, proton pump inhibitors; HR, hazard ratio; NSAIDs, non-steroidal anti-inflammatory drugs; ^†^ Charlson comorbidity index; ^‡^ Adjusted for age, gender, hypertension, hyperlipidemia, Charlson comorbidity index, H2 blocker, NSAIDs and corticosteroid; * *p* < 0.05; ** *p* < 0.01.

**Table 3:** Subgroup analysis of Cox proportional hazard model of CNS infection events in PPI and Non-PPI groups.

	PPI		Non-PPI			
	*N*	No. of CNS Infection Event	*N*	No. of CNS Infection Event	HR	95% CI
Age ^a^						
20–40	2123	2	2005	3	0.59	0.10–3.57
40–65	8557	20	8637	10	2.23 *	1.04–4.76
≥65	5561	14	5599	5	3.23 *	1.16–8.99
Gender ^b^						
Female	7911	16	7912	9	1.98	0.88–4.50
Male	8330	20	8329	9	2.39 *	1.09–5.28
Hypertension ^c^						
No	9842	19	9848	13	1.59	0.78–3.23
Yes	6399	17	6393	5	3.80 **	1.40–10.32
Hyperlipidemia ^d^						
No	13,039	30	13,076	16	2.09 *	1.13–3.83
Yes	3202	6	3165	2	3.09	0.62–15.40
Charlson comorbidity index ^e^						
0	4618	6	4592	4	1.57	0.44–5.59
1	8664	18	8780	8	2.47 *	1.07–5.70
≥2	2959	12	2869	6	2.25	0.84–6.04
H2 Blocker ^f^						
No	12,551	27	12,558	15	2.17 *	1.14–4.15
Yes	3690	9	3683	3	2.34	0.71–7.68
NSAIDs ^g^						
No	14,076	25	13,958	15	1.83	0.96–3.48
Yes	2165	11	2283	3	4.08 *	1.13–14.7
Corticosteroid ^h^						
No	13,873	23	13,784	13	1.92	0.97–3.80
Yes	2368	13	2457	5	2.91 *	1.03–8.2
Immunosuppressant ^i^						
No	16,164	36	16,167	18	2.20 **	1.25–3.89
Yes	77	0	74	0	NA	NA

CNS, central nervous system; PPI, proton pump inhibitors; HR, hazard ratio; NSAIDs, non-steroidal anti-inflammatory drugs; ^a^ Adjusted for gender, H2 Blocker, NSAIDs, and corticosteroid; ^b^ Adjusted for age, hypertension, hyperlipidemia, Charlson comorbidity index, H2 Blocker, NSAIDs, and corticosteroid; ^c^ Adjusted for age, gender, hyperlipidemia, Charlson comorbidity index, H2 Blocker, NSAIDs, and corticosteroid; ^d^ Adjusted for age, gender, hypertension, H2 Blocker, NSAIDs, and corticosteroid; ^e^ Adjusted for age, gender, hypertension, H2 Blocker, NSAIDs, and corticosteroid; ^f^ Adjusted for age, gender, hypertension, hyperlipidemia, Charlson comorbidity index, NSAIDs, and corticosteroid; ^g^ Adjusted for age, gender, hypertension, hyperlipidemia, Charlson comorbidity index, H2 Blocker, and corticosteroid; ^h^ Adjusted for age, gender, hypertension, hyperlipidemia, Charlson comorbidity index, H2 Blocker, and NSAIDs; ^i^ Adjusted for age, gender, hypertension, hyperlipidemia, Charlson comorbidity index, H2 Blocker, NSAIDs, and corticosteroid; NA: not available; * *p* < 0.05; ** *p* < 0.01.

## Supplementary Materials

The following are available online at http://www.mdpi.com/2077-0383/7/9/252/s1, Figure S1: Box plot of distribution of propensity score, Table S1: Cox proportional hazard model of CNS infection events.

## References

[1] Ward R.M., Kearns G.L. (2013). Proton pump inhibitors in pediatrics: Mechanism of action, pharmacokinetics, pharmacogenetics, and pharmacodynamics. Paediatr. Drugs.

[2] Strand D.S., Kim D., Peura D.A. (2017). 25 years of proton pump inhibitors: A comprehensive review. Gut Liver.

[3] Sachs G., Shin J.M., Howden C.W. (2006). Review article: The clinical pharmacology of proton pump inhibitors. Aliment. Pharmacol. Ther..

[4] Corleto V.D., Annibale B., Gibril F., Angeletti S., Serrano J., Venzon D.J., Delle Fave G., Jensen R.T. (2001). Does the widespread use of proton pump inhibitors mask, complicate and/or delay the diagnosis of Zollinger-Ellison syndrome?. Aliment. Pharmacol. Ther..

[5] Altman K.W., Haines G.K., Hammer N.D., Radosevich J.A. (2003). The H^+^/K^+^-Atpase (proton) pump is expressed in human laryngeal submucosal glands. Laryngoscope.

[6] Wang J., Barbuskaite D., Tozzi M., Giannuzzo A., Sorensen C.E., Novak I. (2015). Proton pump inhibitors inhibit pancreatic secretion: Role of gastric and non-gastric H^+^/K^+^-Atpases. PLoS ONE.

[7] Shiraev T.P., Bullen A. (2018). Proton pump inhibitors and cardiovascular events: A systematic review. Heart Lung Circ..

[8] Li T., Xie Y., Al-Aly Z. (2018). The association of proton pump inhibitors and chronic kidney disease: Cause or confounding?. Curr. Opin. Nephrol. Hypertens..

[9] Trifan A., Stanciu C., Girleanu I., Stoica O.C., Singeap A.M., Maxim R., Chiriac S.A., Ciobica A., Boiculese L. (2017). Proton pump inhibitors therapy and risk of clostridium difficile infection: Systematic review and meta-analysis. World J. Gastroenterol..

[10] Hafiz R.A., Wong C., Paynter S., David M., Peeters G. (2018). The risk of community-acquired enteric infection in proton pump inhibitor therapy: Systematic review and meta-analysis. Ann. Pharmacother..

[11] Ho S.W., Tsai M.C., Teng Y.H., Yeh Y.T., Wang Y.H., Yang S.F., Yeh C.B. (2014). Population-based cohort study on the risk of pneumonia in patients with non-traumatic intracranial haemorrhage who use proton pump inhibitors. BMJ Open.

[12] Ho S.W., Teng Y.H., Yang S.F., Yeh H.W., Wang Y.H., Chou M.C., Yeh C.B. (2017). Association of proton pump inhibitors usage with risk of pneumonia in dementia patients. J. Am. Geriatr. Soc..

[13] Fallahzadeh M.K., Borhani Haghighi A., Namazi M.R. (2010). Proton pump inhibitors: Predisposers to alzheimer disease?. J. Clin. Pharm. Ther..

[14] Badiola N., Alcalde V., Pujol A., Munter L.M., Multhaup G., Lleo A., Coma M., Soler-Lopez M., Aloy P. (2013). The proton-pump inhibitor lansoprazole enhances amyloid beta production. PLoS ONE.

[15] Goldstein F.C., Steenland K., Zhao L., Wharton W., Levey A.I., Hajjar I. (2017). Proton pump inhibitors and risk of mild cognitive impairment and dementia. J. Am. Geriatr. Soc..

[16] Tsai C.F., Chen M.H., Wang Y.P., Chu C.J., Huang Y.H., Lin H.C., Hou M.C., Lee F.Y., Su T.P., Lu C.L. (2017). Proton pump inhibitors increase risk for hepatic encephalopathy in patients with cirrhosis in a population study. Gastroenterology.

[17] Riddell J.T., Shuman E.K. (2012). Epidemiology of central nervous system infection. Neuroimaging Clin. N. Am..

[18] Davies N., Thwaites G. (2011). Infections of the nervous system. Pract. Neurol..

[19] Agastya G., West B.C., Callahan J.M. (2000). Omeprazole inhibits phagocytosis and acidification of phagolysosomes of normal human neutrophils in vitro. Immunopharmacol. Immunotoxicol..

[20] Mikawa K., Akamatsu H., Nishina K., Niwa Y. (2001). Effects of pirenzepine, omeprazole, lansoprazole, and rabeprazole on human neutrophil functions. Can. J. Anaesth..

[21] Zedtwitz-Liebenstein K., Wenisch C., Patruta S., Parschalk B., Daxbock F., Graninger W. (2002). Omeprazole treatment diminishes intra-and extracellular neutrophil reactive oxygen production and bactericidal activity. Crit. Care Med..

[22] Garcia-Martinez I., Frances R., Zapater P., Gimenez P., Gomez-Hurtado I., Moratalla A., Lozano-Ruiz B., Bellot P., Gonzalez-Navajas J.M., Such J. (2015). Use of proton pump inhibitors decrease cellular oxidative burst in patients with decompensated cirrhosis. J. Gastroenterol. Hepatol..

[23] Fried M., Siegrist H., Frei R., Froehlich F., Duroux P., Thorens J., Blum A., Bille J., Gonvers J.J., Gyr K. (1994). Duodenal bacterial overgrowth during treatment in outpatients with omeprazole. Gut.

[24] Thorens J., Froehlich F., Schwizer W., Saraga E., Bille J., Gyr K., Duroux P., Nicolet M., Pignatelli B., Blum A.L. (1996). Bacterial overgrowth during treatment with omeprazole compared with cimetidine: A prospective randomised double blind study. Gut.

[25] Adriani K.S., Brouwer M.C., van de Beek D. (2015). Risk factors for community-acquired bacterial meningitis in adults. Neth. J. Med..

[26] Lundbo L.F., Benfield T. (2017). Risk factors for community-acquired bacterial meningitis. Infect. Dis..

[27] Gowin E., Januszkiewicz-Lewandowska D. (2018). Genes and their single nucleotide polymorphism involved in innate immune response in central nervous system in bacterial meningitis: Review of literature data. Inflamm. Res..

[28] Haenisch B., von Holt K., Wiese B., Prokein J., Lange C., Ernst A., Brettschneider C., Konig H.H., Werle J., Weyerer S. (2015). Risk of dementia in elderly patients with the use of proton pump inhibitors. Eur. Arch. Psychiatry Clin. Neurosci..

[29] Wijarnpreecha K., Thongprayoon C., Panjawatanan P., Ungprasert P. (2016). Proton pump inhibitors and risk of dementia. Ann. Transl. Med..

[30] Tai S.Y., Chien C.Y., Wu D.C., Lin K.D., Ho B.L., Chang Y.H., Chang Y.P. (2017). Risk of dementia from proton pump inhibitor use in Asian population: A nationwide cohort study in Taiwan. PLoS ONE.

[31] Ortiz-Guerrero G., Amador-Munoz D., Calderon-Ospina C.A., Lopez-Fuentes D., Nava Mesa M.O. (2018). Proton pump inhibitors and dementia: Physiopathological mechanisms and clinical consequences. Neural Plast..

[32] Hwang I.C., Chang J., Park S.M. (2018). A nationwide population-based cohort study of dementia risk among acid suppressant users. Am. J. Geriatr. Psychiatry.

[33] Batchelor R., Gilmartin J.F., Kemp W., Hopper I., Liew D. (2017). Dementia, cognitive impairment and proton pump inhibitor therapy: A systematic review. J. Gastroenterol. Hepatol..

[34] Liu W., Baker S.S., Trinidad J., Burlingame A.L., Baker R.D., Forte J.G., Virtuoso L.P., Egilmez N.K., Zhu L. (2013). Inhibition of lysosomal enzyme activities by proton pump inhibitors. J. Gastroenterol..

[35] Peddicord T.E., Olsen K.M., Collier D.S. (1999). Effect of omeprazole, lansoprazole, and ranitidine on the DNA synthesis of mononuclear cells. Crit. Care Med..

[36] Ohara T., Arakawa T. (1999). Lansoprazole decreases peripheral blood monocytes and intercellular adhesion molecule-1-positive mononuclear cells. Dig. Dis. Sci..

[37] Van der Hoorn M.M.C., Tett S.E., de Vries O.J., Dobson A.J., Peeters G. (2015). The effect of dose and type of proton pump inhibitor use on risk of fractures and osteoporosis treatment in older Australian women: A prospective cohort study. Bone.

[38] Jo Y., Park E., Ahn S.B., Jo Y.K., Son B., Kim S.H., Park Y.S., Kim H.J. (2015). A proton pump inhibitor’s effect on bone metabolism mediated by osteoclast action in old age: A prospective randomized study. Gut Liver.

[39] Andersen B.N., Johansen P.B., Abrahamsen B. (2016). Proton pump inhibitors and osteoporosis. Curr. Opin. Rheumatol..

[40] Costa-Rodrigues J., Reis S., Teixeira S., Lopes S., Fernandes M.H. (2013). Dose-dependent inhibitory effects of proton pump inhibitors on human osteoclastic and osteoblastic cell activity. FEBS J..

[41] Prause M., Seeliger C., Unger M., Rosado Balmayor E., van Griensven M., Haug A.T. (2015). Pantoprazole decreases cell viability and function of human osteoclasts in vitro. Mediat. Inflamm..

[42] Motegi H., Abe S., Tansho S., Suzuki D., Yamaguchi H., Hoshino E. (2001). Suppressive effect of lansoprazole on anti-Candida activity of murine macrophages. Kansenshogaku Zasshi.

