# Supplementary material for: Association between Proton Pump Inhibitor Use and CNS Infection Risk: A Retrospective Cohort Study
Source: J Clin Med. 2018 Aug 31;7(9):252. doi: 10.3390/jcm7090252 (PMC6162834; doi:10.3390/jcm7090252)
Supplement: Supplementary file 1 [file jcm-07-00252-s001.pdf]

Supplementary Materials: Association between Proton Pump Inhibitor Use and CNS Infection Risk: A Retrospective Cohort Study

Table S1. Cox proportional hazard model of CNS infection events.

	N	No. of CNS Infection Event	Crude HR	95% CI	Adjusted HR †	95% CI
PPI						
No	168,914	129	1		1	
Yes	16,905	39	3.18 **	2.22–4.55	2.32 **	1.55–3.48

PPI, proton pump inhibitors; CNS, central nervous system; † Adjusted for age, gender, hypertension, hyperlipidemia, Charlson comorbidity index, H2 blocker, NSAIDs, and corticosteroid; ** $p < 0.01$.

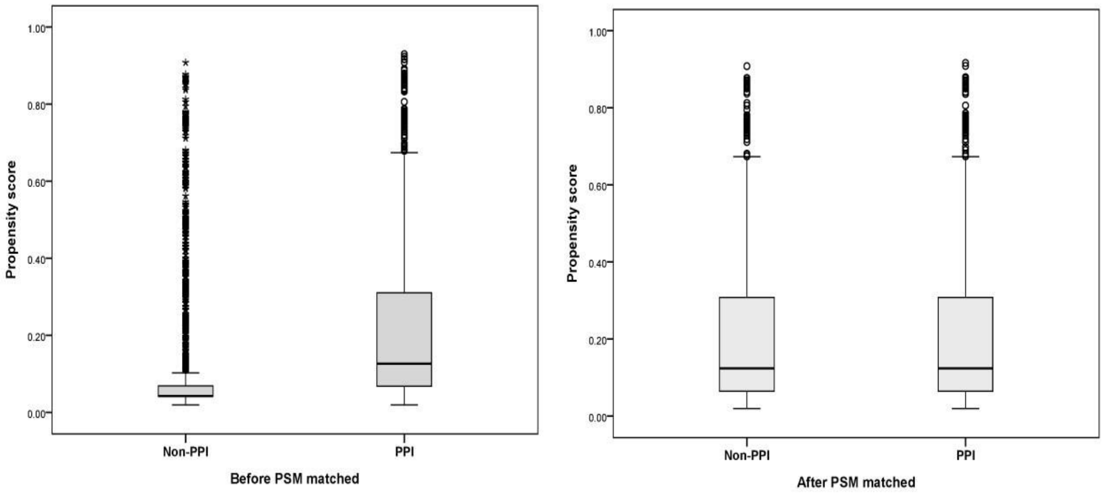

Figure S1. Box plot of distribution of propensity score. PPI, proton pump inhibitors; PSM, propensity score matching.
